# Hepatocarcinoma Induces a Tumor Necrosis Factor-Dependent Kupffer Cell Death Pathway That Favors Its Proliferation Upon Partial Hepatectomy

**DOI:** 10.3389/fonc.2020.547013

**Published:** 2020-10-16

**Authors:** Jean-François Hastir, Sandrine Delbauve, Lionel Larbanoix, Desislava Germanova, Cleo Goyvaerts, Justine Allard, Sophie Laurent, Karine Breckpot, Alain Beschin, Martin Guilliams, Véronique Flamand

**Affiliations:** ^1^Institute for Medical Immunology, Université Libre de Bruxelles, Brussels, Belgium; ^2^Center for Microscopy and Molecular Imaging, Université de Mons, Brussels, Belgium; ^3^Laboratory for Molecular and Cellular Therapy, Vrije Universiteit Brussel, Brussels, Belgium; ^4^Diapath, Center for Microscopy and Molecular Imaging, Université Libre de Bruxelles, Brussels, Belgium; ^5^Laboratory of Cellular and Molecular Immunology, Vrije Universiteit Brussel, Brussels, Belgium; ^6^Myeloid Cell Immunology Laboratory, Vrije Universiteit Brussel, Brussels, Belgium; ^7^Laboratory of Myeloid Cell Ontogeny and Functional Specialization, VIB Center for Inflammation Research, Ghent, Belgium; ^8^Department of Biomedical Molecular Biology, Ghent University, Ghent, Belgium

**Keywords:** Kupffer cells, hepatocellular carcinoma, liver regeneration, partial hepatectomy, cell death, inflammation, tumor necrosis factor-alpha

## Abstract

Partial hepatectomy (PH) is the main treatment for early-stage hepatocellular carcinoma (HCC). Yet, a significant number of patients undergo recursion of the disease that could be linked to the fate of innate immune cells during the liver regeneration process. In this study, using a murine model, we investigated the impact of PH on HCC development by bioluminescence imaging and flow cytometry. While non-resected mice were able to control and reject orthotopic implanted Hepa1-6 hepatocarcinoma cells, resected liver underwent an increased tumoral proliferation. This phenomenon was associated with a PH-induced reduction in the number of liver-resident macrophages, i.e., Kupffer cells (KC). Using a conditional ablation model, KC were proved to participate in Hepa1-6 rejection. We demonstrated that in the absence of Hepa1-6, PH-induced KC number reduction was dependent on tumor necrosis factor-alpha (TNF-α), receptor-interacting protein kinase (RIPK) 3, and caspase-8 activation, whereas interleukin (IL)-6 acted as a KC pro-survival signal. In mice with previous Hepa1-6 encounter, the KC reduction switched toward a TNF-α-RIPK3–caspase-1 activation. Moreover, KC disappearance associated with caspase-1 activity induced the recruitment of monocyte-derived cells that are beneficial for tumor growth, while caspase-8-dependent reduction did not. In conclusion, our study highlights the importance of the TNF-α-dependent death pathway induced in liver macrophages following partial hepatectomy in regulating the antitumoral immune responses.

## Introduction

Primary liver malignancy constitutes one of the most common forms of cancers worldwide associated with a high mortality rate (1). Hepatocellular carcinoma (HCC) accounts for up to 90% of these malignancies (2) and, therefore, constitutes a major health issue. Partial hepatectomy (PH) is a commonly used curative therapy for HCC (3) with good results at early stage (4) and can even lead to better results than transcatheter arterial chemoembolization within patients carefully selected beyond the traditional Milan criteria (5). While hepatic resection is considered as a treatment of choice, a significant number of patients undergo recursion of the disease (6, 7). Recurrence can be either due to the formation of *de novo* tumoral site or to the presence of an ignored cryptic tumoral site not removed during surgery. Relapse constitutes a bad prognostic for the patient and the available therapeutic options might get limited depending on the anatomical location of the tumor, actual liver functions, and general status of the patient. Therefore, the development of strategies aimed at reducing the risk of recursion is a paramount element of the surgery-based approach. Following PH, liver regeneration (compensatory hypertrophy and hyperplasia without restoration of the original anatomical shape) occurs, aiming at re-establishing the numerous physiological functions of the organ. Various signaling molecules and pathways are activated during liver regeneration (including mitogen-activated protein kinases, phosphoinositide 3-kinases, insulin-like growth factor, and hepatocyte growth factor pathways) and participate in the process (8, 9). Yet, major alterations of these pathways are linked with the development and progression of liver cancers (10–12). Immune cells play a key role in driving and participating in the activation of the complex process leading to the compensatory hyperplasia of hepatocytes. Most of the studies on liver regeneration have focused on deciphering the mechanisms leading to hepatocyte proliferation in the absence of pathology. Therefore, the impact that PH has on immune cells and how it affects tumor recurrence are still not fully understood. In a normal context, Kupffer cells (KC) drive the early response to liver partial ablation by producing tumor necrosis factor-alpha (TNF-α) and interleukin (IL)-6 that in turn stimulate hepatocyte proliferation through activation of nuclear factor kappa B (NF-κB) and signal transducer and activator of transcription 3 (STAT3) pathway, respectively (13–16). Both of these cytokines are associated with tumor aggressiveness and metastasis (10, 11). Phosphorylated STAT3 (i.e., activated) has been found in a majority of human HCC, and this activation was associated with tumor aggressiveness and poor prognosis (17). As for NF-κB, its inhibition in different mouse models of HCC was associated with limited tumor development (18). It is therefore expected that tumor cells would be able to use TNF-α and IL-6 signaling to their own advantage. On the other hand, NF-κB's ability to maintain antioxidant defenses can also contribute to reduce liver damage (18), and in diethylnitrosamine-induced HCC, NF-κB participates in the maintenance of hepatocyte survival resulting in limiting cancer development (19). TNF-α is also a cell death inducer and a pro-inflammatory cytokine that can activate immunity. Indeed, signaling through TNFR1 can lead, under specific conditions, to the formation of a protein complex containing RIPK1 and RIPK3, which can either lead to the phosphorylation of mixed lineage kinase-like (MLKL) and the induction of necroptosis or the activation of caspase-8 and subsequent induction of apoptosis (20). Finally, aggression of the liver can lead to monocyte and monocyte-derived cell recruitment. This is notably the case in other liver injury models such as acetaminophen-induced liver fibrosis model where monocyte-derived cells with a different phenotype than KC (referred here as Ly6C^low^ macrophages) can be found and participate in the remodeling of the organ (21). Such population's recruitment following PH and impact on the recurrence phenomenon is also poorly described.

In the present study, we used an *in vivo* mouse model of HCC and PH combined with bioluminescence imaging to study the impact of PH on primary Hepa1-6 HCC development. We demonstrated the protective role of KC in this setup using conditional ablation and further analyzed the *in vivo* mechanisms modifying the innate immune response toward a tumor-favorable environment following PH.

## Materials and Methods

### Mice

Eight- to 12-week-old male C57BL/6 mice were used (ENVIGO, Zeist, Netherlands). IL-6 KO and CCR2 KO mice were purchased from Jackson Laboratory (Bar Harbor, ME). RIPK3 KO mice were provided by Peter Vandenabeele (Inflammation Research Center, VIB, Ghent, Belgium). Myeloid TNF KO mice (TNF^M−KO^ mice; TNFflox/flox LysMcre/cre mice) were provided by Sergei Nedospasov (Engelhardt Institute of Molecular Biology, Russian Academy of Sciences and Lomonosov Moscow State University, Moscow, Russia) and KC-DTR mice were mated in our specific pathogen-free animal facility (Gosselies, Belgium). All animals received humane care according to the criteria outlined in the “Guide for the Care and Use of Laboratory Animals” prepared by the National Academy of Sciences (NIH publication 86-23 revised 1985).

### Hepa1-6 and Hepa1-6-Fluc Cell Lines

Mycoplasma-free Hepa1-6 cells (ATCC) and Hepa1-6-Fluc cells generated through transduction with lentiviral vectors encoding firefly luciferase (transfer plasmid pDUAL_SFFV-Fluc_Ub-puroR) were cultured in Dulbecco's modified Eagle's medium (DMEM/Lonza, BioWhittaker™) supplemented with 10% heat-inactivated fetal bovine serum, 2 mM l-glutamine, 1 mM non-essential amino acids, 100 mM sodium pyruvate, penicillin (10 U/ml)–streptomycin (10 μg/ml), 10–5M 2-ME (Lonza Research Products, Basel, Switzerland), and puromycin (5 μg/ml, Sigma-Aldrich).

### Surgical Procedure for Orthotopic Tumor Implantation and Partial Hepatectomy

Mice were injected with Hepa1-6-Fluc cells 1 week before partial hepatectomy (H+PH group). The control group did not undergo surgery (H group). For flow cytometry experiments, a third group of control mice underwent phantom operation (sham group). Mice were then used either for bioluminescence imaging or flow cytometry experiments. For experiments investigating partial hepatectomy, mice underwent 40% partial hepatectomy and their liver was collected at various time points following surgery.

Under anesthesia (xylazine 50 mg/kg and ketamine 100 mg/kg), a small midline laparotomy was performed on prehydrated (0.9% NaCl, 200 μl) mice. For tumor inoculation, the median lobe of the liver was exposed and injected under the Glisson's capsule with 10^6^ Hepa1-6 cells suspended in 50 μl PBS. For partial hepatectomy, the left lobe of the liver was ligated and resected. Body temperature was maintained at 36.5–37°C during the surgical procedures. The abdominal wall and the skin were sutured separately. Sham-operated mice underwent the same procedure with 50 μl PBS injection and without ligation and resection of the left lobe of the liver.

### KC Depletion

KC-DTR mice were intraperitoneally injected with 2 or 5 ng of diphtheria toxin (Sigma) 7 days after intrahepatic Hepa1-6-Fluc inoculation.

### Bioluminescence Imaging

*In vivo* follow-up was performed after tumor inoculation and carried over the 4 weeks following surgery or phantom operation. Mice were anesthetized with 4% of isoflurane vaporized in 2 L/min O_2_ and then maintained with 2% isoflurane in 0.3 L/min O_2_ per mouse. Before imaging, mice were shaved to decrease the light absorption and scattering of animal fur. Each animal received s.c. 150 mg/kg body weight of a 20-mg/ml solution of D-luciferin in a 20-mg/ml solution in NaCl 0.9% (VivoGlo, Promega). Mice were imaged in a Photon Imager Optima (Biospace Lab, France) that dynamically counted the emitted photons for at least 25 min. Image analysis was performed with M3Vision software (Biospace Lab). ROIs were drawn on the mice abdomen in the liver area and signal intensities were quantified individually for a time lapse of 5 min corresponding to the maximum signal intensity plateau.

### Flow Cytometry

Livers were collected at various time points following resection or phantom operation (24 h, 36 h, 2 days, or 7 days). The liver lobes were weighted and transferred into gentleMACS tubes (Miltenyi Biotec, Leiden, Netherlands) supplemented with RPMI 1640 medium and collagenase A (type III, Worthington Biochemicals, New Jersey, USA) and DNase I (Roche) for one round of the m_liver_01_03 protocol of the gentleMACS dissociator (Miltenyl Biotec). After 20 min at 37°C, tubes completed the m_liver_02_03 protocol of the same dissociator. The obtained suspension was diluted in FACS buffer and passed through a sterile 100-μm filter, centrifuged (1,400 rpm, 7 min at 4°C), and resuspended for 1 min in ammonium–chloride–potassium lysis buffer.

Caspase-8 and caspase-1 activity assays were performed following the manufacturer's protocol. Cells were incubated with FAM-FLICA (Bio-Rad AbD) for 1 h before proceeding to standard extracellular staining. Propidium iodide (2 μl) from the same kit (Bio-Rad AbD) was used for staining 15 min at room temperature prior to standard extracellular staining.

For standard extracellular staining, cells were resuspended and stained in the dark at 4°C for 20 min with polyclonal unconjugated anti-Clec4F antibodies (R&D Systems). Samples were then incubated in the dark at 4°C for 20 min with a mix of antibodies purchased from BD Biosciences (CD45, Ly6G, Ly6C, CD11b, CD11c), eBioscience (F4/80, Tim4), BioLegend (PDCA1), and Invitrogen (secondary antibody for Clec4F detection).

For intracellular staining of p-MLKL (Ser345), cells were fixed using the Foxp3 kit from eBioscience. Cell permeabilization and fixation were run in accordance with the manufacturer's protocol. After washing, cells were incubated at 4°C for 20 min in the dark with a primary p-MLKL (Ser345) (D6E3G) antibody (Cell Signaling Technology). After a washing step, a secondary detection antibody (anti-rabbit IgG FabAlexa Fluor® 488 Cell Signaling Technology) was incubated with the cells at 4°C for 20 min in the dark. For intracellular staining of IL-6 and TNF, cells were incubated with BD GolgiPlug™ (1 μl/ml), phorbol 12-myristate 13-acetate (5 ng/ml), and ionomycin (500 ng/ml) for 4 h at 37°C prior to staining of extracellular markers. After extracellular staining, cells were fixed using the BD Cytofix/Cytoperm™ Fixation/Permeabilization Kit. Experiments were run in accordance with manufacturer's protocol. Cells were incubated at 4°C for 30 min in the dark with anti-TNF, anti-IL6, or control isotype (BD Biosciences). Samples were measured using the BD LSR Fortessa™ (BD Bioscience, Erembodegem, Belgium). The total amount of cells passed for each sample varies from 700,000 to 1,400,000 cells. Data were analyzed using the FlowJo V9.9.6 software (FlowJo, Ashland, USA).

### RNA Purification and Real-Time Reverse Transcription Polymerase Chain Reaction

Liver was collected at various time points following partial hepatectomy or phantom operation (0 min, 30 min, 1 h, 2 h, overnight). RNA was extracted from liver lobes using an EZNA HP Total RNA Kit (Omega Bio-tek, Georgia, USA). Extracted RNA samples were quantified using the NanoDrop™ spectrophotometer and stored at −20°C before being used for reverse transcription quantitative polymerase chain reaction (qPCR). For the quantification of transcripts, reverse transcription and qPCR were performed in a single step using the TaqMan RNA Amplification (Roche Diagnostics) on a Lightcycler 480 apparatus (Roche Diagnostics) with the following conditions: 10 min at 50°C, 10 min at 45°C and 30 s at 95°C, and then 45 cycles of 5 s at 95°C and 30 s at 60°C. For the granulocyte macrophage-colony stimulating factor (GM-CSF) gene, RNA was reverse-transcribed with the transcriptor High fidelity cDNA synthesis (Roche Diagnostics). cDNA was amplified using SYBR green. For individual samples, relative RNA levels (2–ΔΔCt) were determined by comparing a) the cycle thresholds (Ct) for the gene of interest and calibrator gene (ΔCt), Hprt, and b) 2–ΔCt values for the experimental group vs. the reference sample (H group). The sequences of primers and probes are presented in Table 1.

**Table 1:** The sequences of primers and probes.

**Gene**	**Forward**	**Reverse**	**Probe**	**PCR product**
MCP1	CTTCTGGGCCTGCTGTTCA	CCAGCCTACTCATTGGGATCA	CTCGCCAGATGCAGTTAACGCCCC	127 bp
GM-CSF	ACCCGCCTGAAGATATTCG	AGCTGGCTGTCATGTTCAAG	/	69 bp
IL6	GAGGATACCACTCCCAACAGACC	AAGTGCATCATCGTTGTTCATACA	CAGAATTGCCATTGCACAACTCTTTTCTCA	140 bp
TNFα	CAGACCCTCACACTCAGATCA	CACTTGGTGGTTTGCTACGA	TCGAGTGACAAGCCTGTAGCCCA	78 bp
HPRT	GGACCTCTCGAAGTGTTGGAT	CCAACAACAAACTTGTCTGGAA	CAGGCCAGACTTTGTTGGATTTGAA	70 bp

### Histology

Formalin-fixed hepatic lobes of interest were embedded in paraffin. Five-micrometer liver sections were stained with hematoxylin/eosin (HE). Conversion of glass slides into digital data was performed using a NanoZoomer 9200S (Hamamatsu Photonics K.K.). Determination of tumor size was performed on a digital slide using the NDP.view2 software (Hamamatsu Photonics K.K.).

### Statistical Analysis

Statistical comparison between experimental groups was made using GraphPad Prism (GraphPad Software, Inc.). The nature of the test used is described in the figure legends. *P* values less than or equal to 0.05 were considered significant.

## Results

### Partial Hepatectomy Favors the Proliferation of Hepa1-6 Cells in the Liver

**Figure 1:**
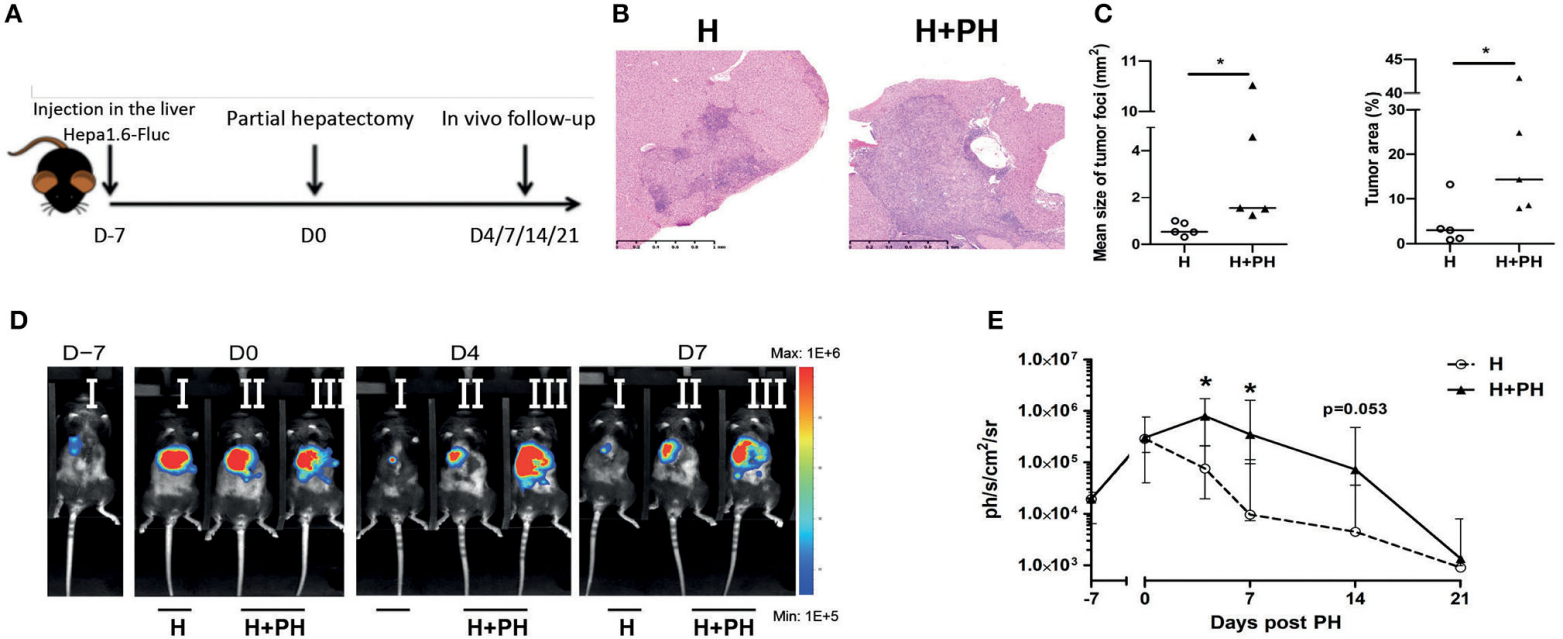
Proliferation of Hepa1-6 cells is increased following partial hepatectomy. **(A)** Experimental procedure used. Mice were injected with Hepa1-6-Fluc cells 1 week before partial hepatectomy (H+PH group). The control group did not undergo surgery (H group). **(B)** Liver histology visualized by HE staining. Representative pictures of the nonresected (H) and resected (H+PH) experimental groups. **(C)** Quantification of tumor burden by mean size of tumor foci (left panel) and percentage of tumor on a slide (right panel) in wild-type animals. **(D)** Representative image of the bioluminescence data generated until 7 days post PH. **(E)** Bioluminescence signal detection over time following partial hepatectomy. Two-tailed Mann–Whitney test, *p < 0.05. Results presented as median and interquartile range (*n* = 7/group) from at least three independent experiments.

In order to evaluate the impact of PH on tumoral development, we used a preclinical model based on the injection of a murine hepatocellular carcinoma cell line (Hepa1-6 cells) in the liver median lobe. One week later, the experimental group of mice underwent PH by resection of their left lobe, accounting for 40% of the total liver mass (H+PH group), and the control group underwent phantom operation (H group) (Figure 1A). Tumor implantation was confirmed by histological observations in wild-type animals. We observed that PH increases the tumoral burden as monitored by the increased median size of tumor foci and higher global size of the tumor (Figures 1B,C). We further quantified the impact of PH on tumoral development by inoculating Hepa1-6 cells expressing the firefly luciferase enzyme (Hepa1-6-Fluc cells) in the liver median lobe, which allowed for *in vivo* follow-up of tumor over time. As described by Sakai et al. (22), the tumoral development of Hepa1-6-Fluc cells in the control group of mice was halted after a first week period of proliferation (Figures 1D,E). Concerning the impact of PH on tumor growth, bioluminescence signal in the PH group was significantly increased 4 and 7 days after PH and underwent a delayed abrogation after 21 days instead of 7 days in the H group of mice (Figures 1D,E), indicating that tumor cell proliferation is uncontrolled during liver regeneration, a process that in a murine model takes about 1 week for completion [(8, 23) and Supplementary Figure 1].

### Partial Hepatectomy Modifies Innate Immune Cell Composition in the Liver

**Figure 2:**
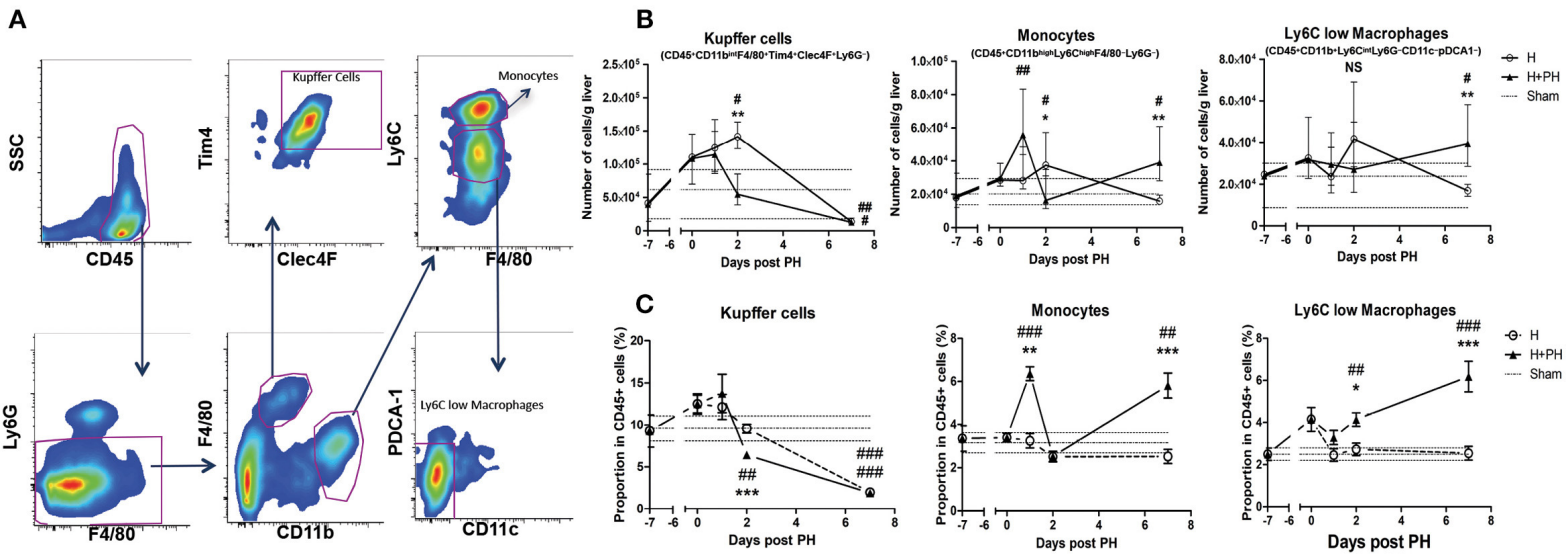
Absolute number and proportion of Kupffer cells among CD45^+^ cells are reduced following PH, while monocytes and Ly6C^low^ macrophages increase after surgery. **(A)** Representative picture of the gating strategies used for the discrimination of Kupffer cells, monocytes, and Ly6C^low^ macrophages. **(B)** Kinetics of the number of Kupffer cells, monocytes, and Ly6C^low^ macrophages following PH in C57BL/6 mice liver. Flow cytometry analyses were done 0, 1, 2, and 7 days following PH (H+PH group) or phantom operation (H group). The sham group was injected with physiological serum and received placebo surgery 7 days later. *p < 0.05, **p < 0.01, Kruskal–Wallis/one-way ANOVA followed by Dunn's *post hoc* test/Bonferroni's multiple comparison test between the H and H+PH groups/^#^*p* < 0.05, ^##p^ < 0.01, Kruskal–Wallis/one-way ANOVA followed by Dunn's *post hoc* test/Bonferroni's multiple comparison test between the sham and H and H+PH groups (*n* = 4–9/time point). Choice of the test was based on the result of the Shapiro–Wilk normality test. Results presented as median and interquartile range from at least three independent experiments. **(C)** Kinetics of the proportion among leukocytes (CD45^+^ cells) of Kupffer cells, monocytes, and Ly6C^low^ macrophages following PH in C57BL/6 mice liver. Flow cytometry analyses were done 0, 1, 2, and 7 days following PH (H+PH group) or phantom operation (H group). The sham group was injected with physiological serum and received placebo surgery 7 days later. *p < 0.05, **p < 0.01, ***p < 0.001, Kruskal–Wallis/one-way ANOVA followed by Dunn's *post hoc* test/Bonferroni's multiple comparison test between the H and H+PH groups/^#^*p* < 0.05, ^##^*p* < 0.01, ^###^*p* < 0.001, Kruskal–Wallis/one-way ANOVA followed by Dunn's *post hoc* test/Bonferroni's multiple comparison test between the sham and H and H+PH groups (*n* = 4–9/time point). Choice of the test was based on the result of the Shapiro–Wilk normality test. NS, not significant.

We next sought to determine the possible causes of the increased tumor proliferation we observed following PH. Since the regeneration process is known to be relying on innate immune cells and that KC are important gatekeepers of liver physiology, we evaluated their absolute numbers and proportion in total leukocyte population (determined by the expression of CD45) using flow cytometry (gating strategies are described in Figure 2A). First, we observed that the number of KC (CD45^+^ CD11b^int^ F4/80^+^ Tim4^+^ Clec4F^+^ Ly6G^−^ cells) progressively increased and reached maximal value 9 days after tumor inoculation in the H group of mice compared with the sham group of mice (placebo treatment). A drastic decrease of KC number was observed at day 2 post PH in the H+PH group compared with the H group (Figure 2B). Then, both groups had significantly lowered KC number compared with the sham group 14 days post tumor inoculation (Figure 2B). Analysis of the proportion of KC in CD45^+^ cells confirmed the reduction observed 2 days after PH (Figure 2C). Yet, the increased absolute number of cells observed in the H group at the same timing did not translate to an increased proportion in CD45^+^ cells, indicating that other CD45^+^ populations were recruited in this experimental condition at that time. The absolute number of monocytes (CD45^+^ CD11b^high^ Ly6C^high^ F4/80^−^ Ly6G^−^ cells) also progressively increased and reached maximal value 9 days after tumor inoculation in the H group compared with the sham and H+PH groups of mice. As for KC on that day, this increased absolute number did not translate to an increase in the proportion in CD45^+^ cells. Two significant increases of monocyte number were observed in the H+PH group at day 1 post PH when compared with the sham group and at day 7 post PH when compared with the H and sham groups. The same type of observations was made in the proportion of CD45^+^ cells with a significant increase observed at day 1 and day 7 post PH (Figures 2B,C). Concerning the Ly6C^low^ macrophages (CD45^+^ CD11b^+^ Ly6C^int^ Ly6G^−^ CD11c^−^ pDCA1^−^), their number increased significantly at day 7 post PH in the H+PH group compared with the H group of mice (Figure 2B). This observation corroborated the one made when analyzing the proportion of these cells in CD45^+^ population. Moreover, a significantly higher proportion of Ly6C^low^ macrophages were also observed as early as day 2 post PH (Figure 2C). In contrast to KC, both numbers of monocytes and Ly6C^low^ macrophages were increased compared with the sham group 7 days post PH.

Taken together, our results demonstrate that PH induced a drop in KC number in tumor-bearing liver and an “earlier” monocyte influx as well as another “delayed” monocyte recruitment that correlates with a late increased number and proportion in CD45^+^ cells of Ly6C^low^ macrophages in the organ.

### KC Depletion Results in an Increased Hepa1-6 Proliferation

**Figure 3:**
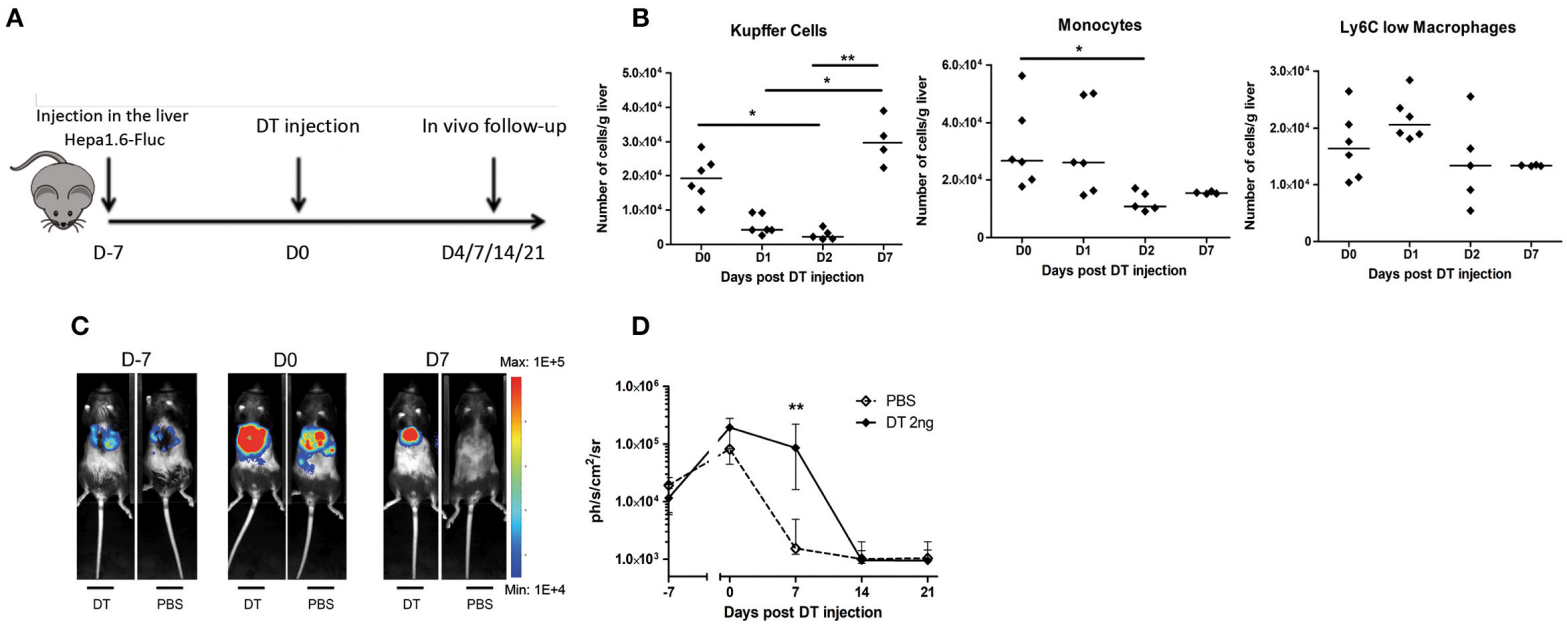
Proliferation of Hepa1-6 cells is increased following Kupffer cell depletion. **(A)** Experimental procedure used. Mice were injected with Hepa1-6-Fluc cells 1 week before intraperitoneal injection of diphtheria toxin (DT 2 ng group) or PBS. **(B)** Kinetics of the absolute number of KC, monocytes, and Ly6C^low^ macrophages in KC-DTR mice after 2-ng DT injection. *p < 0.05, **p < 0.01, Kruskal–Wallis followed by Dunn's *post hoc* test. Results presented as median. **(C)** Representative image of the bioluminescence data generated until 7 days post injection. **(D)** Bioluminescence signal detection over time following injection. Two-tailed Mann–Whitney test, **p < 0.001. Results presented as median and interquartile range (*n* = 7–8/group) from at least three independent experiments.

The reduction of KC observed in mice undergoing PH associated with the increased tumor proliferation raised the possibility that they would be important agents in the antitumoral process. Macrophages are known for their dual role during cancer development, and while resident cells can limit its progression in early stages, tumor-associated macrophages derived from monocytes found at a later time point have an anti-inflammatory “alternatively activated” (M2) phenotype. This is notably known in humans as HCC where later-stage patients with poor prognosis have increased M2 macrophages infiltrating the tumor (24). Nevertheless, PH is a treatment reserved for early-stage patients and it is thus expected that liver-resident KC would limit cancer development. To evaluate the impact of the decreased KC number on tumor growth, we mimic the effect of PH-induced KC disappearance by using KC-DTR mice previously described by Scott et al. (25). This strain has the unique characteristic of being specifically depleted in the KC compartment after diphtheria toxin (DT) injection in a dose-dependent manner (25). We used a single 2-ng injection of DT (Figure 3A) that does not cause side effects on the liver regeneration post PH as evaluated by the liver to body weight ratio in WT mice (Supplementary Figure 2A). That DT dose caused a partial decrease of KC numbers (Supplementary Figure 2B), which went back to pre-injection level 1 week following the injection (Figure 3B). This recovery was associated with a decreased number of monocytes in the organ 2 days post DT injection. No significant modifications in the Ly6C^low^ macrophage compartment could be observed at tested timing (Figure 3B), demonstrating that our model specifically induces modifications in the KC compartment.

Next, we observed a significantly increased proliferation of Hepa1-6-Fluc cells during the first week following the 2-ng DT injection, while PBS-injected mice naturally rejected the tumor (Figures 3C,D), effectively recapitulating the previous observations in mice undergoing liver regeneration (Figure 1). Taken together, our results indicate that the reduction of the absolute number of KC observed in mice undergoing liver regeneration might, at least partially, explain the increased tumoral proliferation observed in this condition, raising the question on the mechanisms leading to KC disappearance.

### TNF and IL-6 Influence KC Survival During Liver Regeneration and Impact the Tumor Proliferation

**Figure 4:**
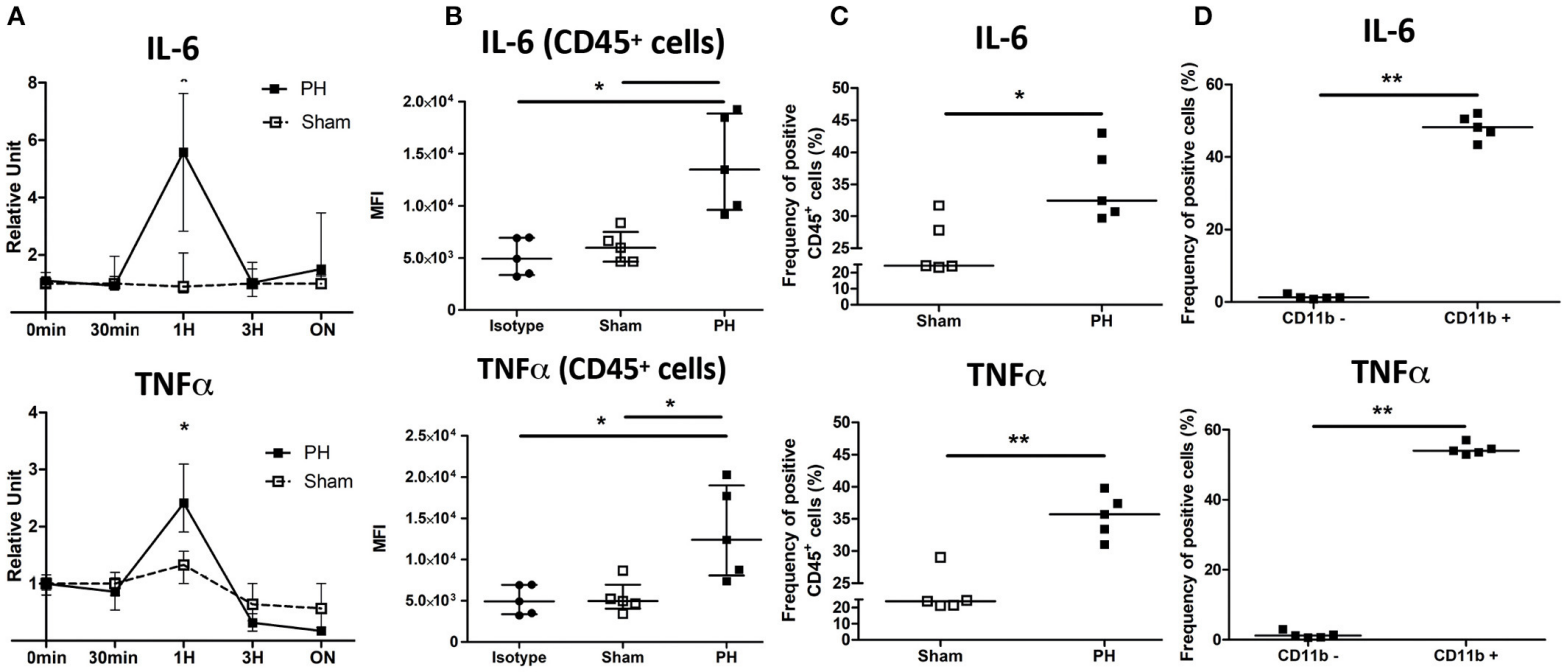
IL-6 and TNF are induced following partial hepatectomy. **(A)** Relative expression in total liver of C57BL/6 mice of IL-6 and TNF over time following PH or phantom sham operation. *p < 0.05, two-tailed Mann–Whitney test (*n* = 3–7/time point). **(B)** Median fluorescence intensity (MFI) of C57BL/6 CD45^+^ cells stained for TNF, IL-6, or control isotype (isotype group) 24 h following partial hepatectomy (PH group) or phantom operation (sham group). *p < 0.05, Kruskal–Wallis followed by Dunn's *post hoc* test. Results presented as median and interquartile range. **(C)** Frequency of CD45^+^ cells producing TNF or IL-6 24 h following partial hepatectomy (PH group) or phantom operation (sham group). Determination of positive gate was based on isotype control staining. Results presented as median. *p < 0.05, **p < 0.01, two-tailed Mann–Whitney test. Results presented as median. **(D)** Frequency of CD11b^−^ cells and CD11b^+^ producing TNF or IL-6 24 h following partial hepatectomy. Cells were firstly gated for CD45 expression. Determination of positive gate was based on isotype control staining. **p < 0.01, two-tailed Mann–Whitney test. Results presented as median.

We next wanted to decipher the mechanism linking the KC number reduction and the increased tumor growth induced upon PH. We observed that the transcript levels of IL-6 and TNF, two major actors of liver regeneration, were significantly increased in the partially resected liver 1 h post surgery (Figure 4A). This increase was also observed at protein level 24 h after surgery. Intracellular staining of IL-6 and TNF revealed a significant increase of the median fluorescence intensity and the proportion of CD45^+^ cells positively stained following PH as compared with the control group (Figures 4B,C). We also observed that following PH, the myeloid compartment of CD45^+^ cells (discriminated on the basis of CD11b expression) had a significantly higher proportion of cells positively stained for both cytokines than the CD11b^**−**^ cells (Figure 4D).

**Figure 5:**
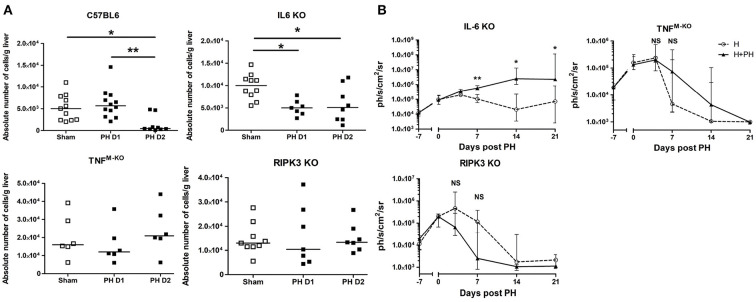
PH-induced Kupffer cell number reduction is regulated by TNF-α and IL-6. **(A)** Absolute number of KC in C57BL/6, IL-6 KO, TNF^M−KO^, and RIPK3 KO mice. Analyses were run at day 1 or 2 following PH. The control sham group underwent phantom operation. **(B)** Bioluminescence signal detection over time following partial hepatectomy in IL-6 KO, TNF^M−KO^, and RIPK3 KO mice. Two-tailed Mann–Whitney test, *p < 0.05, **p < 0.01, NS: non-significant difference (*n* = 7–8/group). All results presented as median and interquartile range from at least three independent experiments.

First, we evaluated the role of IL-6 in the outcome of KC post PH. We observed that IL-6 KO mice displayed a faster decrease in KC numbers 24 h following resection (Figure 5A), whereas WT mice underwent a decrease 2 days post PH. This indicates that IL-6 would act as a KC cytoprotective factor. Moreover, tumoral proliferation was strongly increased in the H+PH group of IL-6 KO mice compared with the H group 7, 14, and 21 days post PH (Figure 5B). Of note, in this strain compared with WT mice, both the H and H+PH groups failed at rejecting the tumor even at day 21, strengthening the role of IL-6 as a major early contributor to KC survival and to the liver protection against tumor proliferation.

Next, we evaluated the role of TNF and the RIPK3 cell death-associated signaling molecule. To do this, we ran PH experiments in TNF^flox/flox^LysM^Cre/WT^ mice (TNF^M−KO^ mice with exclusive *Tnf* gene deletion in lysozyme M-expressing myeloid cells like monocytes, neutrophils, and macrophages) and in RIPK3 KO mice. We observed that the PH-induced KC disappearance observed in C57BL/6 mice was abrogated in TNF^M−KO^ mice and in RIPK3 KO mice (Figure 5B). Interestingly, we observed that the TNF^M−KO^ and RIPK3 KO strains showed no increased Hepa1-6-Fluc cell proliferation after PH (Figure 5C).

### Liver Resection Induces a RIPK3-Dependent Activation of Caspase-8 in KC

**Figure 6:**
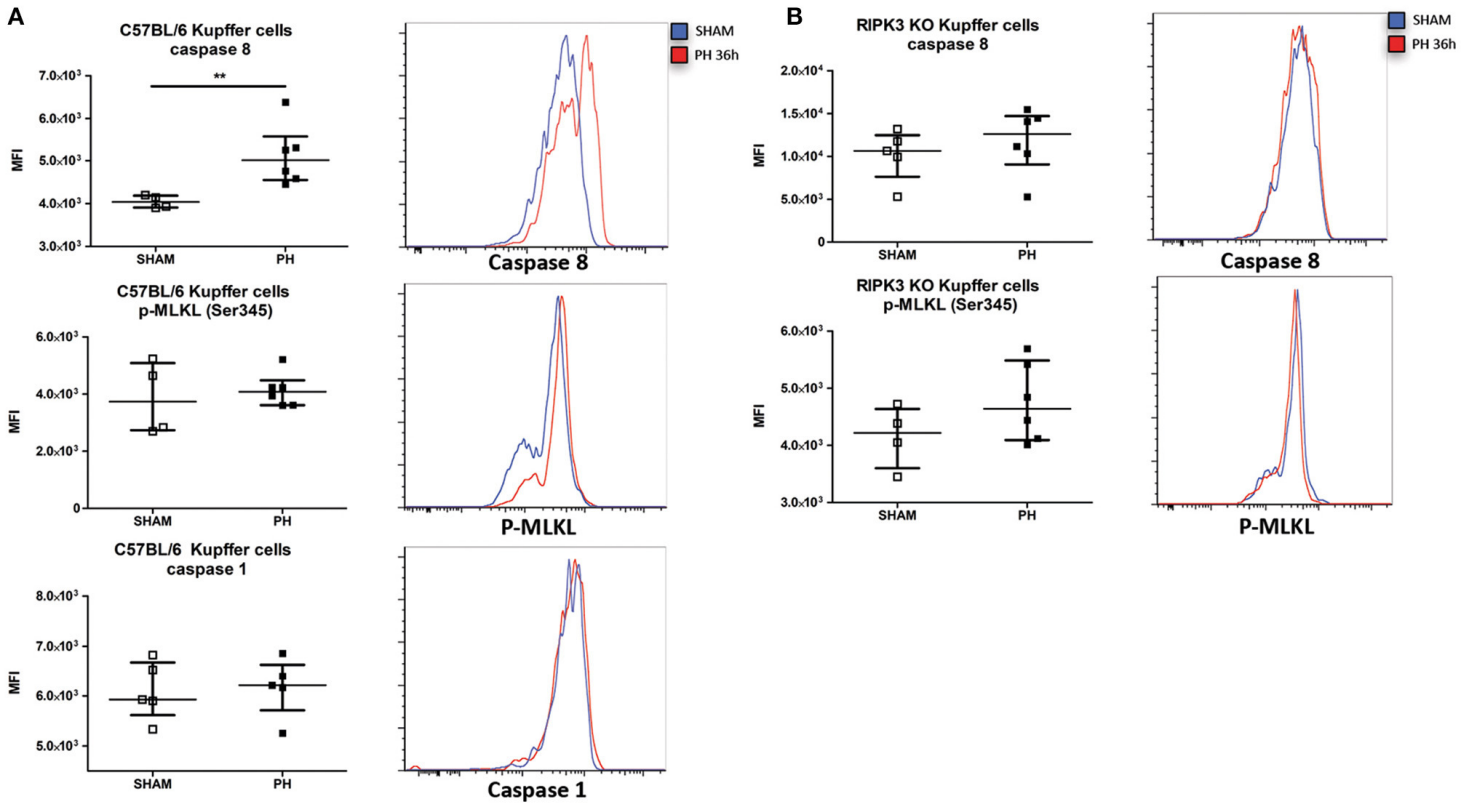
PH induces a RIPK3-dependent apoptosis of Kupffer cells. (**A**, upper panel) Median fluorescence intensity (MFI) of C57BL/6 KC stained with fluorescent-labeled inhibitor of caspase-8 36 h post PH and representative histogram of the fluorescence intensity. **p < 0.01, two-tailed Mann–Whitney test. (**A**, middle panel) MFI of C57BL/6 KC stained for Ser345 phosphorylated MLKL (p-MLKL Ser345) 36 h post PH and representative histogram of the fluorescence intensity. (**A**, lower panel) MFI of C57BL/6 KC stained with labeled inhibitor of caspase-1 36 h post PH and representative histogram of the fluorescence intensity. (**B**, upper panel) MFI of RIPK3 KO KC stained with fluorescent-labeled inhibitor of caspase-8 36 h post PH and representative histogram of the fluorescence intensity. Results presented median and interquartile range. (**B**, lower panel) MFI of RIPK3 KO KC stained for p-MLKL Ser345 36 h post PH and representative histogram of the fluorescence intensity. All results presented as median and interquartile range from at least three independent experiments.

We next sought to determine the mechanism responsible for the TNF-dependent KC reduction following PH (i.e., KC apoptosis via caspase-8, necroptosis via phosphorylation of MLKL, or pyroptosis via caspase-1). In assays determining the functional ability of the protein, we detected an increased caspase-8 activity but no increased levels of caspase-1 and p-MLKL (Ser345) in WT mice KC following PH (Figure 6A). Neither increased caspase-8 activation nor increased level of p-MLKL (Ser345) was observed in RIPK3 KO KC under the same condition (Figure 6B). Taken together, our results support the idea that following PH, KC undergo a TNF/RIPK3-dependent apoptosis resulting in a reduction of their number in the organ.

### Tumor Encounter Switches TNF/RIPK3-Dependent Activation of Caspase-8 to TNF/RIPK3-Dependent Activation of Caspase-1 in KC Following PH

**Figure 7:**
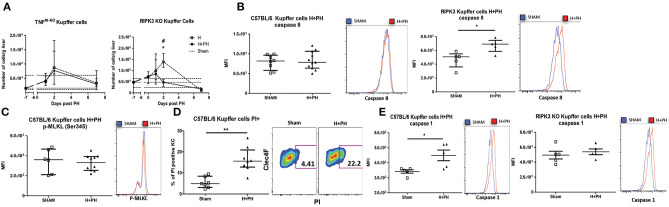
Tumor encounter switches caspase-8 activation to caspase-1 activation in KC following partial hepatectomy. **(A)** Kinetics of KC following PH in TNF^M−KO^ and RIPK3 KO mice liver. Flow cytometry analyses were done 0, 1, 2, and 7 days following PH (H+PH group) or phantom operation (H group). The sham group was injected with physiological serum and received placebo surgery 7 days later. *p < 0.05, Kruskal–Wallis followed by Dunn's *post hoc* test between the H and H+PH groups/#*p* < 0.05, Kruskal–Wallis followed by Dunn's *post hoc* test between the sham and other experimental groups (*n* = 5–9/time point). Test chosen based on the result of the Shapiro–Wilk normality test. **(B)** MFI of C57BL/6 or RIPK3 KO KC from mice injected with Hepa1-6 cells and stained with fluorescent-labeled inhibitor of caspase-8 36 h post PH or phantom operation (sham) and representative histogram of the fluorescence intensity. *p < 0.05, two-tailed Mann–Whitney test. **(C)** MFI of KC of C57BL/6 from mice injected with Hepa1-6 cells, stained for p-MLKL Ser345 36 h post PH and representative histogram of the fluorescence intensity. **(D)** Percentage of positive KC for propidium staining 36 h post PH and representative dot plot of the staining. **p < 0.01, two-tailed Mann–Whitney test. **(E)** MFI of C57BL/6 or RIPK3 KO KC from mice injected with Hepa1-6 cells and stained with fluorescent-labeled inhibitor of caspase-1 36 h post PH and representative histogram of the fluorescence intensity. *p < 0.05, two-tailed Mann–Whitney test. All results presented as median and interquartile range from at least three independent experiments.

We further evaluated the role of TNF/RIPK3 in the fate of KC post PH in tumor-inoculated mice. While PH-induced KC disappearance was still abrogated in H+PH TNF^M−KO^ mouse, we observed a sharp drop of KC in H+PH RIPK3 KO mice 2 days post PH (Figure 7A), in opposition with KC survival in tumor-free RIPK3 KO mice after PH (Figure 5A). This suggested that Hepa1-6 had an impact on the cell death signaling pathway. Indeed, in contrast with KC from WT mice undergoing PH (Figure 6A), KC from the H+PH group of WT mice were no longer associated with an increase in caspase-8 activity nor increased level of p-MLKL (Ser345) post PH (Figures 7B,C). Yet, an increased fraction of KC was positively stained with propidium iodide at the same timing (Figure 7D), indicating that they were undergoing cell death. We further observed that KC from H+PH WT mice displayed an increased caspase-1 activity, a hallmark of pyroptosis (Figure 7E). We further linked inflammasome activation with TNF/RIPK3 signaling by demonstrating that KC from RIPK3 KO mice were, in contrast to WT mice, positive for caspase-8 but not for caspase-1 activity under the same conditions (Figures 7B,E). Taken together, our results support that tumor encounter modifies the TNF/RIPK3-dependent induction of caspase-8 activity to TNF/RIPK3-dependent caspase-1 activity after PH. This suggests an effective switch from apoptosis to pyroptosis induction in KC.

### KC Death Pathway Influences the Recruitment of Ly6C^low^ Macrophages and Monocytes Promoting Tumor Growth

**Figure 8:**
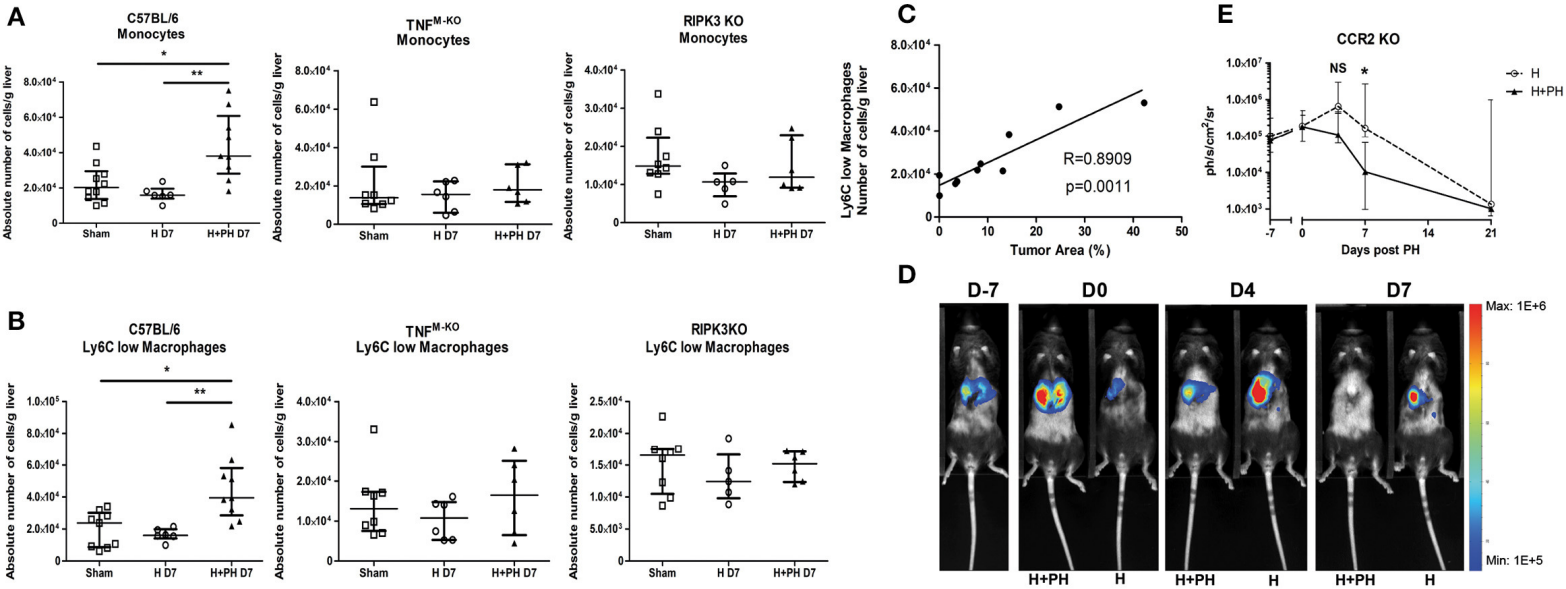
PH induces a recruitment of monocyte-derived cells favorable for tumor proliferation that correlates with the differential death pathway activated in KC. **(A**, **B)** Absolute number of monocytes and Ly6C^low^ macrophages in C57BL/6, TNF^M−KO^, and RIPK3 KO mice injected with Hepa1-6. Flow cytometry analyses were done 7 days following PH (H+PH group) or phantom operation (H group). The sham group was injected with physiological serum and received placebo surgery 7 days later. *p < 0.05, **p < 0.01, Kruskal–Wallis followed by Dunn's *post hoc* test. **(C)** Pearson correlation analysis between absolute number of Ly6C^low^ macrophages and size of the tumor. Absolute number of cell was determined by flow cytometry and tumor size by histological analysis (percentage of tumor on a slide) from wild-type animals at day 7. Pearson correlation (*R*-value) and statistical significance (*p*-value) are displayed. **(D)** Representative image of the bioluminescence data generated until 7 days post PH in CCR2 KO mice. **(E)** Bioluminescence signal detection over time following partial hepatectomy. Two-tailed Mann–Whitney test, *p < 0.05. Results presented as median and interquartile range (*n* = 5/group) from at least three independent experiments. NS, not significant.

The protection against the PH-induced tumoral proliferation in RIPK3 KO mice remained unexplained from the previous experiments. We noticed that in contrast to WT mice, RIPK3 KO mice and TNF^M−KO^ mice did not display any increase in monocytes nor Ly6C^low^ macrophages 7 days post PH (Figures 8A,B). Taking into account the previously shown correlation between the presence of monocytes and Ly6C^low^ macrophages, we first demonstrated that PH induces the expression of CCL2 (a chemoattractant for monocyte) and GM-CSF in the early hours following PH in tumor-free wild-type mice (Supplementary Figure 3A). We then followed the kinetics of Ly6C^low^ macrophages and monocytes in PH wild-type mice (Supplementary Figure 3B). Recruitment of Ly6C^low^ macrophages in wild-type strain was especially striking on day 2 post PH. Moreover, CCR2 KO liver had an effective reduction of both monocytes and Ly6C^low^ macrophages (Supplementary Figure 3B) with no modifications of the KC compartment kinetics post PH.

Zigmond et al. (21) described the restorative and remodeling aspect of similar Ly6C^low^ macrophages, which raised the hypothesis that these cells might be beneficial for tumor development. This idea was strengthened by the finding of a strong correlation (Pearson correlation, *R* = 0.8909/*p* = 0.0011) between the amount of Ly6C^low^ macrophages in the liver and the size of the tumor assessed by histological analysis at the same timing (Figure 8C). Moreover, the CCR2 KO strain showed no significantly increased tumor proliferation following PH (Figures 8D,E), and the protection observed in RIPK3 KO strain can, at least partially, be attributed to the absence of recruitment of these cells.

## Discussion

Partial hepatectomy gives a good overall survival chance in patients carefully selected (5, 26). Yet, tumor recurrence constitutes a major problem for this approach with complications in nearly 70% of the cases at 5 years (7, 26). Complications can arise notably from occulted tumor sites, a scenario that our model reproduces. Clinical and experimental studies have suggested that liver regeneration following surgical resection facilitates tumor growth following a surgery procedure (27–29), a concept that our results support.

While the importance of T-cell immunity in the rejection of the Hepa1-6 cell line was described previously (22), an observation that we confirmed in our model (data not shown), our results clearly demonstrate a role for KC, and therefore the innate immunity component, in tumor rejection. This places KC at the intersection between the induction of liver regeneration and antitumoral responses.

Based on our flow cytometry observations and KC-DTR bioluminescence experiments, we suggest that during the early stages of tumoral development, the absence of KC induced by PH participates in increased tumoral proliferation, while maintenance of their number (and presumably their inflammatory factors) allows for accelerated rejection of the tumor cells. Interestingly, we could observe a reduction in the amount of monocytes in KC-DTR mice 2 days following DT injection. As already demonstrated in the KC-DTR strain, following DT injection, monocytes are recruited and differentiate in KC. Stellate cells and endothelial cells orchestrate this phenomenon (30). We therefore expect the same differentiation mechanism to be responsible for our observation.

The reduction in KC number following PH seemed to replicate the diminution observed in bacterial and ischemia–reperfusion injury models. In particular, the importance of KC necroptosis induced by phagocytosis of bacteria, in generating antimicrobial response production of CCL2 and IL-1β, has underlined the death pathway induced in macrophages as an element regulating inflammation (31). While our results ruled out necroptosis as the reason for KC reduction since no increase of phosphorylated MLKL level could be seen, we firstly described the TNF-α/RIPK3-dependent activation of caspase-8 occurring in KC upon PH. Presumably, activation of the complex IIb downstream of TNFR1 mediates KC apoptosis between days 1 and 2 following PH (Figure 8). TNF induces IL-6 production by KC and triggers hepatocyte proliferation via STAT3 activation during the regenerative process (8). While our results demonstrate that IL-6 is also a crucial factor for KC maintenance and survival in early stages of the regenerative process as well as for tumor clearance, complex IIb formation and subsequent apoptosis might be seen as a way to regulate KC activity following PH and avoid oversignalization via complex I, NF-κB activation, continuous inflammation, and damages in the regenerating organ.

**Figure 9:**
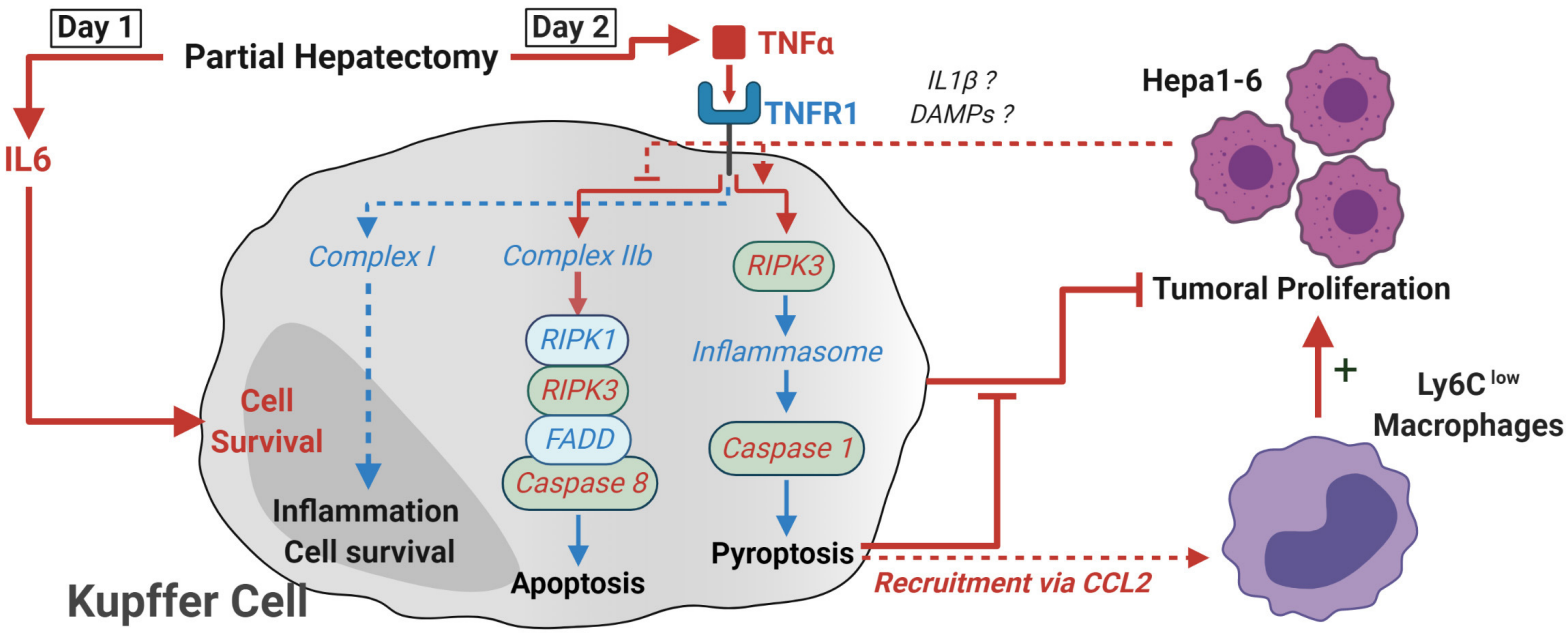
Proposed model of how TNF-α and RIPK3 induce KC death following PH. Following PH, IL-6 acts on KC as a pro-survival signal. In the absence of tumor, TNF signaling leads to the activation of complex IIb and the apoptosis of KC through activation of caspase-8 between day 1 and day 2. The presence of tumor cells within the organ leads to a switch in the activation of cell death pathways and the RIPK3-dependent activation of caspase-1, probably through the activation of inflammasome macromolecular complexes. This KC death was linked with a CCL2-dependent recruitment of Ly6C^low^ macrophages. This latter population was shown to favor tumor development, while KC maintenance accelerated antitumoral responses. Critical elements demonstrated in the paper are described with red arrows and fonts, while deduction from the literature is in blue.

Our study also shows for the first time that the concomitant growth of tumoral cells effectively switches caspase-8 to caspase-1 activation in KC. While these results suggest an induction of pyroptosis in these cells after PH, detection of cleaved Gasdermin D (a pore-forming protein that is activated after cleavage by caspase-1) within KC would be necessary to confirm this idea. Nevertheless, this activation of cell death mechanisms within KC following PH alongside the effective switch we observed and the importance this switch has on the recruitment of monocytes and monocyte-derived cells beneficial for the tumor had up until now never been documented in this context. Based on our results and published literature, we therefore propose the model presented in Figure 9.

The switch we observed was fully dependent on TNF and RIPK3 signaling since TNF^M−KO^ mice did not show a reduction in KC number and RIPK3 KO KC exhibited increased caspase-8 over caspase-1 activity. RIPK3 is able to activate NLR family pyrin domain containing 3 (NLRP3) inflammasome independently of MLKL presence in bone marrow-derived macrophages (32), and a similar mechanism might be occurring in KC. In dendritic cells, caspase-8 activity was shown to regulate RIPK3-dependent activation of the NLRP3 inflammasome, and suppression of caspase-8 activity favored pyroptosis induction (33). The fact that we observed caspase-8 activity but no caspase-1 induction in KC following PH in animals without tumor might hint toward an analogous regulation in KC. We can only speculate on how the switch from apoptosis to pyroptosis is made. Inflammasome assembly and activation mechanism is still a subject of debate especially for NLRP3. Yet, the current model proposes two distinct signals to be necessary (34): the priming signal being transmitted via TLRs, IL-1R, TNFR, or NOD2 and the second signal depending on Ca2^+^ signaling, K^+^ efflux, changes in cell volume, or rupture of lysosomes or reactive oxygen species (ROS). Tumor proliferation, the inflammation generated against it via IL-1β secretion or liberation of DAMPs from dying cells, which are potent TLR ligands, might be participating in the process. Nevertheless, differential death pathway activation in KC leads to different recruitment of monocytes and monocyte-derived macrophages beneficial for tumor proliferation as the data from CCR2 KO mice confirmed. Even though the first clinical trials using carlumab (CNTO888), a human anti-CCL2 antibody, had disappointing results (35, 36) (despite overall low toxicity), modulation of the CCL2–CCR2 axis seems to be a promising way to alleviate the risk of recurrence following PH.

Monocytes are known for their vascular remodeling abilities, and in acute APAP-induced liver injury, monocyte-derived Ly6C^low^ macrophages, with a similar phenotype to the cells we observed, were shown to be important elements for the resolution of inflammation and to have wound healing and tissue remodeling abilities (21). Moreover, in the subcutaneous tumor development model, monocyte-derived CD11b^+^ MHCII^−^ Ly6C^int^ cells infiltrating the tumor were shown to suppress T-cell proliferation and to have important proangiogenic abilities (37). While those models differ from ours, the close phenotype and the same cellular origin of those cells might be giving a hint toward their protumoral activity in our observations. Since RIPK3 KO KC died from apoptosis and no monocyte nor Ly6C^low^ macrophage recruitment was observed, it is reasonable to think that upon inflammation induced by pyroptosis of KC, circulating monocytes are recruited and differentiate into the organ in cells phenotypically different from KC, while a less inflammatory cell death (apoptosis) would not lead to such recruitment and differentiation. These observations might help in understanding the clinical problems faced in tumor embolization protocol and the re-revascularization of the tumor site and overall tumor growth (38).

The dependence of KC death on TNF signaling raises the possibility for the development of strategies aiming at enhancing tumor rejection. The use of anti-TNF antibody treatment is standard nowadays for rheumatoid arthritis and is also of interest for a variety of other autoimmune disease such as Crohn's disease, psoriasis, ulcerative colitis, and ankylosing spondylitis (39). Yet, this approach has not always been efficient, as shown in multiple sclerosis (40), or even dangerous for patients, as demonstrated in clinical trials for heart failure (41, 42). Based on the protection against tumor proliferation observed in TNF^M−KO^ mice and since TNF signalization is also associated with ischemia–reperfusion injury (another liver-damaging reaction occurring in humans following PH due to a surgical procedure), it seems rational to hypothesize a potential beneficial impact of anti-TNF treatment in patients following PH. Yet, caution should be taken when transposing our results in humans, and choosing the right balance between liver regeneration and tumor rejection might be complex. This would be probably achieved by careful selection of the timing at which the treatment would be implemented. The background on which tumor has developed might also be of importance when assessing the effect of anti-TNF treatment since HCC develops mainly in the liver with a previous inflammatory environment due to pathogen (HBV/HCV) or excessive alcohol consumption (alcoholic liver cirrhosis) (43). Other than anti-TNF treatment, specific macrophage inhibitor of pyroptosis might be just as relevant in our specific case. In conclusion, our work highlights the necessity for a comprehensive multidisciplinary treatment approach following PH in order to reduce the risk of complications occurring after surgery.

## Data Availability Statement

The raw data supporting the conclusions of this article will be made available by the authors, without undue reservation.

## Ethics Statement

The animal study was reviewed and approved by Ethics committee of Biopole ULB Charleroi.

## Author Contributions

J-FH and VF contributed to the concept, design of the research contributed, and writing of the manuscript. J-FH and SD performed the experiments and procedures. LL and SL performed the bioluminescence imaging. DG provided expertise to set up the PH model. CG and KB generated the Hepa1-6-Fluc cell line. JA performed the histology. MG and AB provided the genetically modified mice. All authors contributed to the article and approved the submitted version.

## Conflict of Interest

The authors declare that the research was conducted in the absence of any commercial or financial relationships that could be construed as a potential conflict of interest.

## Abbreviations

KC: Kupffer cells
TNF: Tumor necrosis factor-α
RIPK3: Receptor-interacting protein 3 kinase
PH: partial hepatectomy
H: hepatectomy.
