# Supplementary material for: Hepatocarcinoma Induces a Tumor Necrosis Factor-Dependent Kupffer Cell Death Pathway That Favors Its Proliferation Upon Partial Hepatectomy
Source: Front Oncol. 2020 Oct 16;10:547013. doi: 10.3389/fonc.2020.547013 (PMC7597592; doi:10.3389/fonc.2020.547013)
Supplement: Supplementary file 1 [file Data_Sheet_1.PDF]

Supplementary Material

Supplementary Figure 1: Liver regeneration occurs in one week following PH

(A). Liver mass to body ratio over time of C57BL/6mice following PH. Ratio was calculated as followed: *Ratio=(M Liver/M total)/(M liver Sham/(M total Sham) ×100.* Were "M liver" is the mass in gram of the liver and "M total" the total mass of the animal.

Supplementary Figure 2: 2ng diphtheria toxin injection induces a non liver toxic partial reduction of Kupffer cells number

(A). Liver mass to body ratio of C57BL/6mice and KC-DTR mice receiving 0/2/5ng of DT prior to resection. Measurements were done one week following PH or Sham intervention. Ratio were calcutated as followed : *Ratio=(M Liver/M total)/(M liver Sham/(M total Sham) ×100.* Were "M liver" is the mass in gram of the liver and "M total" the total mass of the animal. *p< 0,05 ***p<0,001 Kruskall-Wallis followed by Dunn's post hoc test. Results presented as median and interquartile range **(B).** Proportion of KCs amongst $CD45^{+}$ cells 24h post administration of various DT doses. *p< 0,05 **p<0,01 ***p<0,001 One-way ANOVA followed by Bonferroni's multiple comparison test. Results presented as mean.

Supplementary Figure 3: Ly6C low macrophages are derived from monocytes recruited via CCL2.

(A). Relative expression in total liver of C57BL/6 mice of GM-CSF and CCL2 over time following PH or phantom sham operation. *p< 0,05 **p<0,01 two-tailed Mann-Whitney test. Results presented as median and interquartile range (n=3-6/timepoint). **(B)**. Kinetics of the absolute number of **KCs**, **Monocytes** and **Ly6C low Macrophages** in **C57BL/6** or **CCR2 KO** mice after partial hepatectomy. *p< 0,05 **p<0,01 ***p<0,01 two-tailed Mann-Whitney test. Results presented as median and interquartile range (n=7-12/timepoint).
