# Supplementary figures and images for: Hepatocarcinoma Induces a Tumor Necrosis Factor-Dependent Kupffer Cell Death Pathway That Favors Its Proliferation Upon Partial Hepatectomy
Source: Front Oncol. 2020 Oct 16;10:547013. doi: 10.3389/fonc.2020.547013 (PMC7597592; doi:10.3389/fonc.2020.547013)

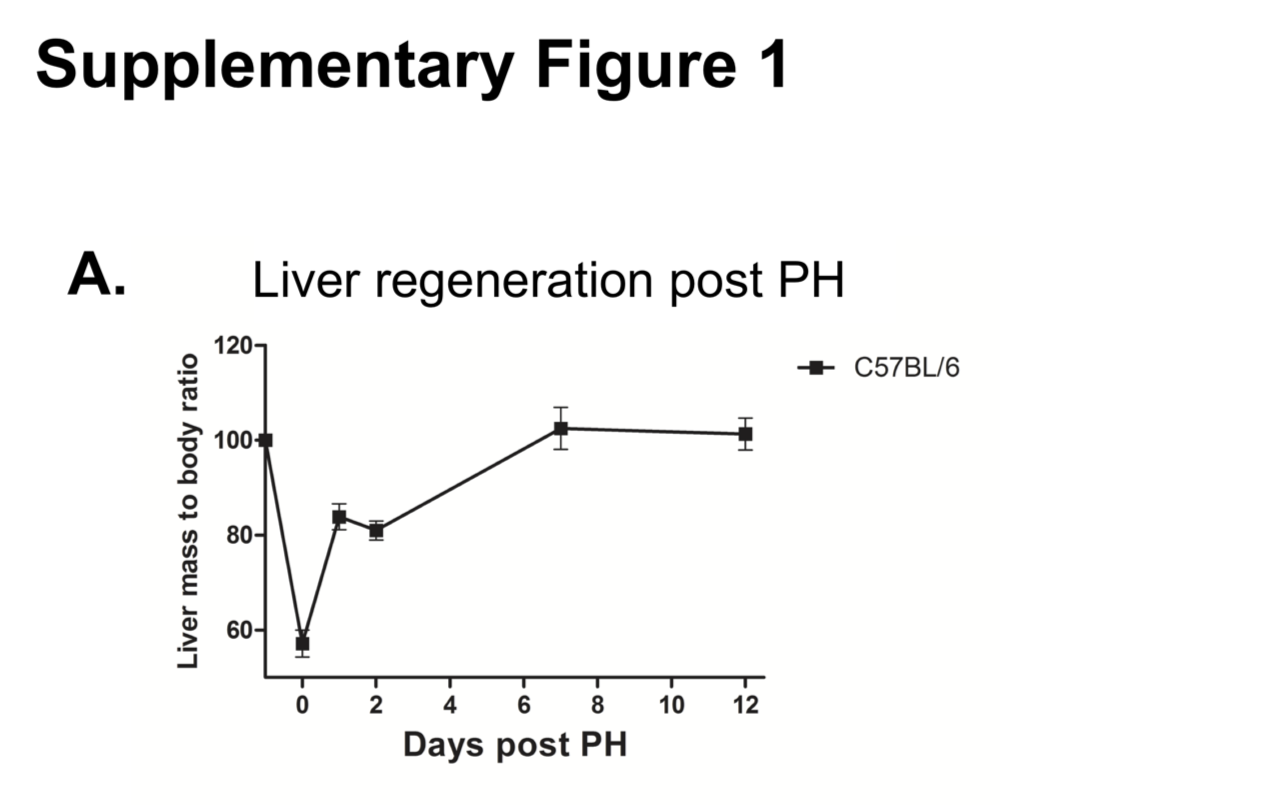

Supplement: Supplementary file 2 [file Image_1.TIFF]

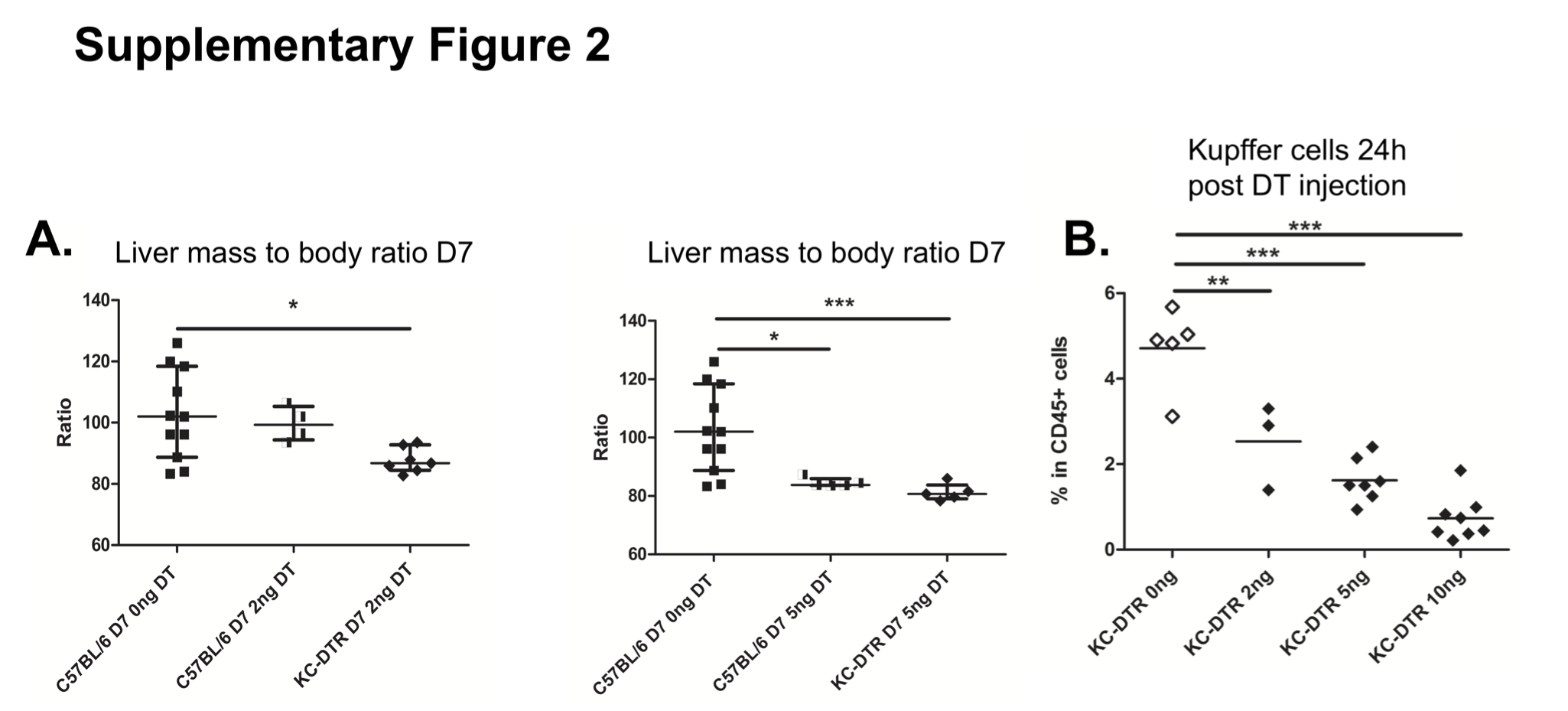

Supplement: Supplementary file 3 [file Image_2.TIFF]

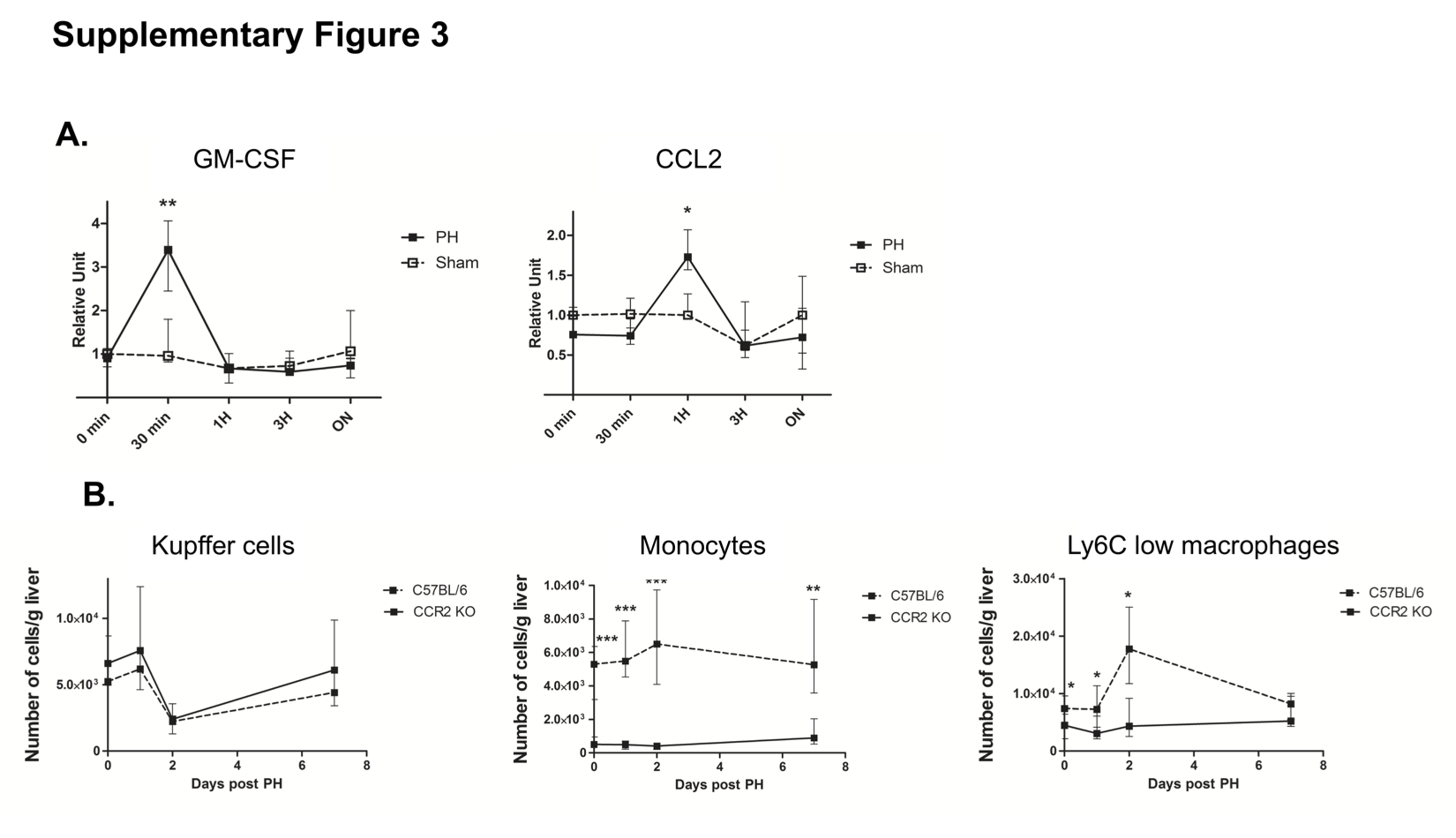

Supplement: Supplementary file 4 [file Image_3.TIFF]
